# Syphilitic Arthritis

**Published:** 1907-10-19

**Authors:** Leonard Gamgee

**Affiliations:** Assistant Surgeon to the General Hospital; Surgeon to the Children's Hospital, Birmingham.


### Hospital Clinics.

### SYPHILITIC ARTHRITIS.

By LEONARD GAMGEE, F.R.C.S., Assistant Surgeon to the General Hospital; Surgeon to the Children's Hospital, Birmingham.

Lancereaux, in his classical "Treatise on Syphilis," gives a short account of the literature relating to syphilitic affections of the joints. Hunter never saw constitutional syphilis attack the joints, but one of his commentators, Babington, admits that syphilitic diseases of the joints exist, and that they occur in both the secondary and the tertiary stages of the disease. However, Richter was the first to describe at all clearly the diseases of the joints due to syphilis. He described two varieties:—(1) The synovial variety, in which the synovial membrane is primarily affected; (2) the bony form, in which the articular ends of the bone are the seat of the disease.

Richter's classification holds good in the main at the present day, but his first variety can be further divided into several sub-classes; while, as a sub-variety of his second form, may be included the epiphysitis so commonly met with in cases of hereditary syphilis.

A more complete clinical classification of syphilitic diseases of the joints is as follows:—

I. In secondary syphilis:—Synovitis, characterised by effusion of fluid and affection of the larger joints. This is usually symmetrical.

II. In tertiary syphilis:—(1) A diffuse thickening of the synovial membranes, resembling in many respects tubercular synovitis, and often referred to as syphilitic white swelling. (2) Synovial thickening accompanied by the formation of isolated gummata in the peri-synovial tissues. (3) An osteitis of the articular ends of the bones, accompanied by more or less effusion of fluid into the joint.

III. In hereditary syphilis:—(1) Subacute inflammation of the epiphyses, generally affecting several of the long bones at the same time and occurring particularly in infancy. (2) Diffuse thickening of the synovial membrane resembling II. (1).

### The Synovitis of Secondary Syphilis.

In my experience synovitis is a very uncommon complication of secondary syphilis. The larger joints, more especially the knees and elbows, are most often affected, and the condition is symmetrical. The signs are those of a subacute effusion into the joint, the synovial membrane not being appreciably thickened; and the patient complains of pain in the joints and some limitation of movement. As a rule this form of syphilitic synovitis occurs at the height of the secondary stage, while the skin rash is well out. Under the internal administration of mercury the effusion soon disappears, leaving behind no stiffness or interference with the functions of the joints. A case in point is the following:—

Case I.—In 1901 a man, aged 30, who was under my treatment at the General Hospital for secondary syphilis, complained on one of his visits of swelling of both elbow-joints. At the time he had a profuse roseola and a papular rash; in fact, his secondary symptoms were at their height. Both elbow-joints were swollen, there being obviously an effusion of fluid into each synovial cavity. The skin over the joints was not reddened; movement, though free, caused some degree of pain. The synovial membrane was not thickened to a perceptible degree, nor was there any thickening of the articular ends of the bones. There was no effusion into, or pain in, any other joints. The patient stated that the elbows had been swollen for ten days. The patient was already taking grs. v. of grey powder a day, and the dose was not increased on account of the synovitis, nor were the joints fixed in splints. I saw the man again in a fortnight's time, and found that the effusion had completely disappeared, and that the movements of the elbows were perfectly free and painless.

The synovitis in this case was evidently due to syphilis; it was symmetrical; it came on when the skin rash was profuse, and it disappeared under the ordinary treatment by mercury.

### Diffuse Synovial Thickening Due to Syphilis.

This, somtimes referred to as syphilitic white swelling, occurs both in the acquired and the hereditary forms of the disease. It is of especial interest, because in many respects it closely resembles tubercular synovitis, a mistake in diagnosis being by no means rare. In my experience this is the most common form of syphilitic joint disease, and the knee is the joint most often affected. The symptoms are as follows:—The synovial membrane is diffusely and uniformly thickened, and presents the pulpy feeling so characteristic of tubercular synovitis. There is occasionally some effusion of fluid, but not to a great amount. The synovitis is often symmetrical; it comes on quietly, runs a chronic course, and, though improvement under anti-syphilitic treatment is marked, there is a tendency to relapse. One of the most important points about this form of synovitis is the freedom of movement and the comparative absence of pain. As regards this latter point, however, there are exceptions; but as regards the freedom of movement of the joint I do not remember to have seen any exceptions, and I regard this as a factor of great value in the differential diagnosis from tubercle.

### Illustrative Cases.

As an illustration of this form of syphilitic synovitis may be quoted:—

Case II.—A. B., a boy aged 11 years, was under my care at the Children's Hospital in 1903. He complained of swelling of both knee-joints and of some aching pain in the knees on walking. There was a diffuse and considerable, but perfectly uniform thickening of the synovial membrane of both knees. No fluid in the joint or swelling of the bone ends could be detected. The patient complained of some pain, not severe, especially felt on walking, yet passive movement of the joints was quite free and did not seem to aggravate the pain. The muscles of both thighs seemed to be wasted; the skin over the affected joints was normal in appearance. The symptoms had been present for three months, in the right knee three weeks longer than the left. The patient was referred to me as having tubercular disease of both knee-joints; but against the accuracy of this diagnosis were to be placed the facts that the disease was symmetrical (for, after all, though tubercular disease of the knee is sufficiently common, yet tubercle does not often affect both knees within a month of one another), that passive movement was free and that it did not aggravate the pain. Moreover, the patient had been kept at rest, yet the condition had progressed all the time. A most important point about the case was the presence of a diffuse thickening of the shaft of the left tibia. This had been present for a month and had come on without apparent cause. The bone was painful, markedly more so at night.

No evidence of syphilis could be obtained by questioning the mother, and no other syphilitic manifestations could be found about the boy himself, except the thickening of the shaft of the tibia. The symmetry of the joint affection, the freedom of movement, and the thickening of the tibia being taken into consideration, the diagnosis made was that of chronic synovial thickening due to hereditary syphilis. The patient was ordered a mixture containing grs. iv. of potassium iodide with ♏ xxx. of liquor. hydrarg. perchlor. to be taken three times a day, the doses to be gradually increased, instructions being given that the boy was to spend most of the day on the couch, but to walk for short distances provided the pain was not severe. From the outset of treatment improvement commenced, the pain soon disappearing and the swelling subsiding. At the end of two months the joints were perfectly normal. The progress of this patient towards recovery was uninterrupted and rapid and, so far as I have been able to find out, no relapse has occurred. That in this variety of synovitis relapses do occur is illustrated by—

Case III.—C. H., a boy aged 18, was under my care in 1895. He had ulceration of the face, necrosis of the nasal bones, old kerato-iritis, and osteitis of the shaft of the right tibia. The boy was treated with iodide of potassium and mercury, and his symptoms rapidly improved. Four months after this I saw him again, and he was then complaining of swelling of the left knee of two months' duration. There was a pulpy, uniform, diffuse thickening of the synovial membrane with a small quantity of fluid in the joint. The bone ends were not thickened. The patient was able to walk without a limp and the movements of the knee were perfectly free and painless. After two months of treatment with iodide of potassium and mercury the swelling disappeared, but it recurred in an exactly similar form six weeks later. The swelling and pain disappeared under treatment and the joint remained sound for the next three years. Since then trace of the patient has been lost.

As has been above stated, this form of syphilitic synovitis may occur in either the hereditary or the acquired disease, but so far as the records of my own cases go it is most common in hereditary syphilis. In fact, I do not remember to have met with a case of synovitis of this kind due to acquired syphilis. Another instance of this form of the disease occurring in hereditary syphilis is the following:—

Case IV.—J. S., a boy aged nine years, has recently been under my care at the General Hospital. In November 1906 he was brought up complaining of pain and some swelling in the left knee. The symptoms had been present for six weeks. There was a pulpy, diffuse uniform thickening of the synovial membrane, but there was no effusion of fluid detectable. The movements of the joint were free, but there was some aching pain complained of. There was no history of injury and there were no signs about the patient to suggest that he suffered from hereditary syphilis.

The diagnosis of tubercular synovitis was made and the joint was fixed on a Thomas knee-splint. There was no improvement under this treatment. In February 1907 the boy came up again. The left knee was *in statu quo*, and the condition of the right knee was now exactly similar. Moreover, at this time the patient had distinct interstitial keratitis. The diagnosis was, therefore, revised, and it was thought that the condition was most probably that of syphilitic synovitis. In order to test the accuracy or otherwise of this latter diagnosis, the Thomas's splint was discontinued, and increasing doses of mercury and iodide of potassium ordered. Under this treatment the pain disappeared rapidly and the swelling steadily, though slowly. The boy was seen on May 27, 1907. He then had no pain in the knees, either when at rest or on walking; the movements of the joints were perfect; there was no swelling about the left knee, but there was still a little thickening of the synovial membrane of the right knee. This was a case in which the condition was first thought to be tubercular, for the synovial membrane exactly resembled that of an early tubercular synovitis. As time went on it seemed well to revise this diagnosis for three reasons: (1) Treatment by rest had resulted in no diminution at all of the amount of swelling; (2) the other knee had become involved in an exactly similar manner; (3) interstitial keratitis had appeared. The result of treatment has proved conclusively that the case was one of syphilitic synovitis.

### Syphilitic Synovitis Accompanied by the Formation of Gummata.

Accompanying a greater or less degree of general thickening of the synovial membrane may be the formation of isolated gummatous nodules in the peri-synovial tissues. As a rule the surface of the synovial membrane looking towards the interior of the joint is not affected, and the gummata form in the fibrous and peri-synovial tissues of the joint.

As the gummata soften they tend to make their way to and to burst on the skin surface, without opening the interior of the joint. This is not invariably the case, for a peri-synovial gumma may in its softening open both on the skin surface and into the interior of the joint. This condition is exemplified by—

Case V.—A man aged 36, came under my care at the General Hospital in February 1897, complaining of swelling of the right knee. The synovial membrane was diffusely thickened, and in the upper part of the swelling was a thickened, indurated patch an inch and a half long by an inch broad. The patch was deeply seated, but was not adherent to the bone. The skin, which was dusky red in colour, could not be quite freely moved over it. To the inner side of the patella was a similar isolated thickened area, while the head of the fibula was distinctly enlarged. The patient had no pain, either when at rest or on moving the joint, while active and passive movements were free and unrestricted. The swelling had been present for six months. The patient stated that at first the skin over the isolated areas of thickening was free and of natural colour, and that it had only recently become bound down and discoloured. The condition had been treated by fixation of the knee in a leather case for two months before the patient came to hospital, but without any benefit. The man denied having had syphilis, but on his prepuce was a scar, due, he said, to a sore which he had many years previously. The freedom from pain and the unrestricted movements were striking features of the case, while the isolated swellings, slowly softening with the skin becoming adherent, resembled gummata more than anything. The provisional diagnosis was that of syphilitic synovitis with peri-synovial gummata, and iodide of potassium was ordered. Under this treatment the swellings, both the general swelling of the synovial membrane and the isolated thickenings, disappeared.

### An Old Description.

Lancereaux, in his "Treatise on Syphilis," gives details of a post-mortem examination of a patient who had multiple syphilitic lesions including arthritis of both knee-joints. "Each knee-joint contained some yellowish, somewhat turbid serum. The synovial membranes, thickened and at the same time injected, were studded with several small pseudo-membraneous deposits.. . . . The articular surface of the left external condyle was eroded and, as it were, ulcerated at one point. . . . On the right side a part of the ligamentum patellæ, the fatty cushion behind, the synovial bursa, and all the fibrous tissues attached around the tibia, are changed into a uniform greyish-yellow elastic mass about an inch and a half thick at the median line. This mass resembled in many respects the morbid products met with in the liver, being, like them, formed by a gummy deposit."

As Lancereaux says, this case shows that the sub-synovial cellular tissue and the fibrous tissue were the seat of the neoplasm, which did not differ either in consistence, colour, or in histological composition from the syphilitic products of the subcutaneous cellular tissue.

### Syphilitic Arthritis in Which the Bone Ends are the Chief Seat of the Disease.

As with tubercle, so with syphilis, the principal seat of an arthritis may be in the articular ends of the bones rather than in the synovial membrane. The condition is really a syphilitic osteitis of the articular ends of one or more of the bones entering into the joint. Accompanying this, in the cases I have seen, has been more or less diffuse thickening of the synovial membrane. This form of syphilitic arthritis differs from those already described by having pain as a most prominent symptom, the pain being of the deeply-seated, aching character with nocturnal exacerbations so typical of chronic bone inflammation. The movements of the joint are free and cause little pain, a characteristic most important for purposes of diagnosis. A clinical picture of this variety of syphilitic arthritis is afforded by—

Case VI.—A man, aged 43, was under my care in 1895. He had had primary syphilis in 1879, and in 1893 his right elbow was swollen, but this swelling subsided. Four months before I saw him his right elbow had again become swollen and painful, and had remained so ever since. The pain was severe at night; but during the day it was so slight that he had been able to continue his work as a labourer, a fact which says a good deal for the painlessness of the joint movements. He had been treated for rheumatism, but with no resulting improvement. When I saw him the right elbow was much swollen. There was regular and uniform swelling of the lower end of the humerus and the upper end of the tibia, while the synovial membrane was thickened diffusely. The skin over the joint was normal in appearance. There was no muscular wasting. The movements, both active and passive, were free and perfectly painless, but the joint could not be flexed or extended to quite the full extent. The diagnosis seemed to rest between tubercular arthritis, osteo-arthritis, and syphilitic arthritis. Had the arthritis been tubercular, with so much accompanying involvement both of bone and synovial membrane, there must have been considerable pain and limitation of movement, and there would certainly have been a marked amount of muscular wasting. Again, there was none of the rigidity and pain on first moving the joint, which is usually met with in cases of osteo-arthritis. Taking the patient's history and symptoms together, the diagnosis made was that of syphilitic arthritis and osteitis. The only treatment ordered was potassium iodide, no splint being applied. The pain and synovial thickening rapidly disappeared, and the enlargement of the bone ends also greatly diminished, though it did not entirely disappear.

Epiphysitis affecting several of the long bones at the same time in infants suffering from hereditary syphilis has been included, in the classification at the commencement of this note, among syphilitic joint affections for the purpose of giving a complete list. More accurately the condition should be included among syphilitic bone diseases, and hence a description of the condition is not included here.

